# The Tonoplast-Localized Sucrose Transporter in *Populus* (PtaSUT4) Regulates Whole-Plant Water Relations, Responses to Water Stress, and Photosynthesis

**DOI:** 10.1371/journal.pone.0044467

**Published:** 2012-08-31

**Authors:** Christopher J. Frost, Batbayar Nyamdari, Chung-Jui Tsai, Scott A. Harding

**Affiliations:** Warnell School of Forestry and Natural Resources and Department of Genetics, University of Georgia, Athens, Georgia, United States of America; Kansas State University, United States of America

## Abstract

The *Populus* sucrose (Suc) transporter 4 (PtaSUT4), like its orthologs in other plant taxa, is tonoplast localized and thought to mediate Suc export from the vacuole into the cytosol. In source leaves of *Populus*, *SUT4* is the predominantly expressed gene family member, with transcript levels several times higher than those of plasma membrane SUTs. A hypothesis is advanced that SUT4-mediated tonoplast sucrose fluxes contribute to the regulation of osmotic gradients between cellular compartments, with the potential to mediate both sink provisioning and drought tolerance in *Populus*. Here, we describe the effects of *PtaSUT4-*RNA interference (RNAi) on sucrose levels and raffinose family oligosaccharides (RFO) induction, photosynthesis, and water uptake, retention and loss during acute and chronic drought stresses. Under normal water-replete growing conditions, SUT4-RNAi plants had generally higher shoot water contents than wild-type plants. In response to soil drying during a short-term, acute drought, RNAi plants exhibited reduced rates of water uptake and delayed wilting relative to wild-type plants. SUT4-RNAi plants had larger leaf areas and lower photosynthesis rates than wild-type plants under well-watered, but not under chronic water-limiting conditions. Moreover, the magnitude of shoot water content, height growth, and photosynthesis responses to contrasting soil moisture regimes was greater in RNAi than wild-type plants. The concentrations of stress-responsive RFOs increased in wild-type plants but were unaffected in SUT4-RNAi plants under chronically dry conditions. We discuss a model in which the subcellular compartmentalization of sucrose mediated by PtaSUT4 is regulated in response to both sink demand and plant water status in *Populus*.

## Introduction

Water is essential for plant growth, development, nutrient and solute transport among tissues, turgor maintenance, and photosynthesis. A number of solutes–including sucrose (Suc) and galactinol (Gol)–play key roles in regulating water balance in plants [1], [2]. Stress imposed on plants by limited water availability affects all aspects of plant development [3] and survival [4], including the ability to cope with other abiotic and biotic stress [5]. As many agricultural areas of the world experience changing rainfall patterns and limited water availability [6], crops as well as undomesticated plants face the challenge of coping with less water [7].

Suc is often the predominant sugar in the vascular transport stream of temperate plant species [8]. Suc movement and concentrations are regulated by the Suc transporter (SUT) transmembrane family of proteins [9], [10]. Suc enters the phloem for long-distance transport both symplastically via plasmodesmatal connections and apoplastically via SWEET-mediated export from mesophyll cells, followed by SUT-mediated uptake into phloem companion cells [11], [12]. The phloem-specific expression of high affinity Group II plasma membrane SUTs is consistent with roles in apoplastic companion cell loading and subsequent recovery of Suc that escapes during phloem transport [9], [13], [14].

Suc is also trafficked across the tonoplast membrane by group IV vacuolar SUTs found so far in Poaceae [15], Salicaceae [16], Brassicaceae [17], Solanaceae [18] and Curcurbitaceae [19]. All tonoplast-localized SUTs identified to date function as SUT/H^+^ symporters that regulate Suc movement from the vacuole lumen to the cytosol [19]–[22]. Such movement facilitates the diurnal shuttling of Suc between the cytosol and vacuole [23], though organ- and tissue-specific dynamics of Group IV tonoplast SUTs vary among plant taxa [15], [24]–[27]. In herbaceous dicots where Suc is loaded apoplastically, Group II plasma membrane SUTs are the predominantly expressed members in source leaves [9]. In the woody perennial *Populus*, expression of Group II SUTs is strongest in stem tissues [16], which is more consistent with recovery of Suc leaked during long-distance transport. Expression of the Group IV tonoplast SUT (*PtaSUT4*) exceeds that of the Group II SUT orthologs in source leaves, which implies that export and long-distance Suc transport in *Populus* is at least partly controlled by PtaSUT4-mediated Suc sequestration within the vacuole [16]. The true mode of phloem loading in *Populus* remains unclarified. On the basis of comparatively high Suc levels in source leaves, and high plasmodesmatal connectivity, phloem ‘loading’ has been postulated to occur by a diffusional gradient through the symplast in *Populus* and related genera [28]. However, a potentially decisive role for plasma membrane SUTs in long distance transport of Suc in species exhibiting high plasmodesmatal connectivity has also been demonstrated [29]. Because Suc is both a metabolite and an osmolyte, and is abundant in *Populus* tissues, its subcellular partitioning is likely to have repercussions for cellular hydrodynamics as well as for source leaf photosynthesis and sink leaf provisioning. Previous characterization of PtaSUT4-RNAi plants revealed increased Suc concentrations in source leaves consistent with impaired Suc export from vacuoles [16]. Here, we report several effects of altered Suc compartmentalization on water uptake and sequestration, source leaf photosynthesis, and source leaf concentrations of water stress-related carbohydrates such as the raffinose (Raf) family oligosaccharides (RFOs).

## Methods

### Plant Material and Growth

Transgenic RNAi lines of poplar (*Populus tremula x P. alba*; clone 717-1B4) with reduced expression of *PtaSUT4* were described previously [16]. Two of the RNAi lines, G and F, with ∼6% and ∼12% residual expression in well-watered plants (see Results), respectively, were selected for the current experiments [16]. Single-node cuttings were grown in perlite under periodic mist until rooted, and then transferred to 4 gallon tree pots (Hummert International, Earth City, MO) containing commercial soil mixture (Fafard 3B, Fafard, Agawam, MA) supplemented with Osmocote (15-9-12 NPK 4-month release; Scotts, Marysville, OH). For the experiments, plants were grown to approximately 1.0–1.5 m in height with daily watering and supplemental lighting as necessary. Stem height and diameter measurements were used to determine growth rates. Diameter measurements were an average of two measurements perpendicular to one another taken from the base (∼3 cm above the soil surface) and at the internodes of leaf plastochron index (LPI) 5 and 20 [30].

### Drought Treatments

The effects of SUT4-RNAi on whole-plant water dynamics were determined by exposing potted saplings to either a short term, acute drought, or a longer term, less severe, chronic drought treatment. The acute water stress was achieved by withholding water from randomly assigned pots of RNAi line G or wild type until the onset of wilt. Soil tensiometers (EC-5, Decagon, Pullman, WA) and data logger (ProCheck, Decagon) were used to monitor changes in soil relative water content (SRWC). One potentiometer was used per pot and the tip was placed at a depth of ∼15 cm below the surface of the soil. We determined from preliminary experiments that the water content of saturated potting mix was ∼0.35–0.40 m^3^/m^3^ (35–40% SRWC) and that turgor loss (i.e., wilt) symptoms began to appear at ∼0.05–0.08 m^3^/m^3^ (5–8% SRWC; Figure S1). Onset of wilt was marked by a downward tilting of leaves from LPI 3 to LPI 6, while the apex region and older source leaves remained turgid. At this stage, turgor was easily restored by watering. With continued absence of water, wilt symptoms propagated down the stem, followed closely by apical drooping and the onset of irreversible leaf collapse. This provided a benchmark by which to monitor the progress of the experimental treatments and anticipate the onset of wilt in subsequent experiments manipulating soil moisture availability.

Two independent experimental trials were conducted with at least n = 6 plants for each treatment group in each trial. Upon initiation of an acute drought stress trial, all plants were watered to saturate the soil. Control plants were then watered daily as normal, while those experiencing acute drought received no water. We harvested all plants in these drought experiments to determine biomass allocation patterns. Biomass from each plant was partitioned into the foliage (including petiole), main stem, and roots. The stem was further partitioned into wood and bark. Roots were washed thoroughly with water to remove soil. All plant parts were oven-dried for 48 hours. For wood and bark, fresh and dry weights were measured to determine moisture concentrations (g water/g FW×100%).

For the chronic drought treatment, rooted cuttings (6–8 replicates per genotype) were potted into the commercial potting soil mix as described above. Following ∼10 days of acclimation to the potting matrix under well-watered conditions, plants were randomly assigned to ‘well-watered’ (25–40% SRWC) or ‘water-limiting’ (8–15% SRWC; minimally sustaining) soil moisture regimes. These SRWC ranges were maintained by careful hand watering and tensiometer monitoring for the majority of their growth (∼2 months) until harvest. Growth measurements were obtained weekly after an initial adjustment period. When plants reached approximately 1–1.4 m in height, gas exchange measurements were taken on LPI 10 (a mature source leaf). A second mature source leaf (LPI 15) was harvested into liquid nitrogen for metabolite analysis. Fresh and dry weights were determined for source leaf (LPI 10), bark, and wood samples, and leaves were photographed to determine leaf area using SigmaScan 3.5 (Systat Software, San Jose, CA) relative to the standard curve for each photograph (Figure S2). From this, we determined water concentrations (g water/g FW×100%) of the tissues and specific leaf area.

### Metabolite Analysis

Mature source leaves (LPI 15), and a limited set of xylem and bark/phloem tissues from well-watered plants were pulverized individually with a mortar and pestle under liquid N_2_ and lyophilized (FreeZone 2.5, Labconco, Kansas City, Missouri). Ten mg of the lyophilized powder was extracted twice with 700 µl methanol:water:chloroform (40%:27%:33%) containing internal standards (ribitol and 2-methoxybenzoic acid; Sigma-Aldrich, St. Louis, MO). The aqueous phase of each round of extractions was pooled and evaporated to dryness (Centrivap Mobile System, Labconco). Samples were resuspended in 40% MeOH:H_2_0 with brief sonication, and then brought to 10% MeOH:H_2_O in the presence of ∼25 mg of Advanta (Applied Separations, Allentown, PA) which was used to trap abundant phenolic metabolites that were not the focus of this investigation. The resulting mixture of small polar metabolites was analyzed by Gas Chromatography-Mass Spectrometry (GC-MS) as described in Jeong et al. [31]. A sub-sample of each extract was transferred to a glass micro-insert and dried in the centrivap. Samples were methoximated with a methoxyamine hydrochloride/pyridine solution (20 mg/ml; Sigma-Aldrich) containing retention index markers (pentadecane, eicosane, pentacosane, and triacontane) [32], and silyated with N-Methyl-N-(trimethylsilyl) trifluoroacetamide (MSTFA; Sigma-Aldrich, St. Louis, MO). Derivitized samples were injected (1 µl per sample) into an Agilent 7890A GC in splitless mode with an inlet temperature of 250°C. Metabolites were resolved on a DB-5MS column (30 m length, 0.25 mm diameter, with a built-in 10 m DuraGuard pre-column) with a flow of 1.12 ml/min, and average velocity of 26.86 cm/sec. Thermal ramping initiated at 80°C for 1 min, ramped 20°C/min to 200°C, then 10°C/min to 310°C with a 6.5 min hold at 310°C. Metabolites were detected using an Agilent 5975C MS with source and quadrupole mass filter temperature setting of 230°C and 150°C, respectively. Mass spectra were collected in scanning ion mode (*m/z* 50 and 500) in ChemStation (Agilent Technologies) and deconvoluted using AnalyzerPro (SpectralWorks, Runcom, UK). Putative peak identities were assigned based on the NIST08 [33], Fiehnlib (Agilent Technologies, [34]), and in-house mass spectral libraries. Compound matching between samples was based on AnalyzerPro name calls, retention index and mass spectral profiles [31]. Retention index and spectral match factor thresholds for all identified metabolites were <1% and >85%, respectively. A select set of samples was analyzed for chlorophyll content according to Porra et al. [35].

### Gas Exchange Measurements

Leaf gas exchange characteristics of wild type and transgenic lines F and G were determined on (1) a dedicated set of plants grown under well-watered conditions and (2) plants subject to long-term contrasting soil moisture regimes (chronic drought experiment) using a Licor LI-6400XS (LiCor, Lincoln, NE). For the dedicated set (1), we generated light curves to determine maximum photosynthetic rate (A_max_), stomatal conductance (Gs), transpiration (E), quantum yield, light compensation point, and basal respiration rate. Light intensities (2000,1500, 1000, 500, 200, 100, 50, 20, 0 µmol/m^2^/sec) were maintained with wait times between 90 and 200 sec at each light intensity depending on ΔCO_2_ and ΔH_2_O stability slopes <1 for 15 seconds. For the chronic drought experiment (2), A_max_, Gs, E, and internal leaf CO_2_ concentration (C_i_) were determined at a single, saturating light intensity of 1500 µmol/m^2^/s with a minimum hold time of 90 sec, followed by manually observing the slope of ΔCO_2_ (using the real-time graphing function in the LI6400) to assess stability between 90 and 120 sec. Gas exchange measurements were taken between approximately 10 am and 3 pm. Preliminary experiments indicated that time of day did not influence light-assisted photosynthesis measurements, and the amount of variance in photosynthesis explained by the time of collection was 1.8%. There was more time-dependent variance in transpiration and stomatal conductance (6.8% and 13.7%, respectively), but was not different between wild-type and SUT4-RNAi genotypes (*p*∼0.76).

### Quantitative PCR

RNA was extracted from source leaves via a modified CTAB method [36], and subjected to cDNA synthesis as described previously [16]. PtaSUT4 transcript levels were determined using qPCR, and analyzed by the 2^−ΔCt^ method with geometric mean of two housekeeping genes (actin, elongation factor 1b) as described in detail in Tsai et al. [37]. PCR parameters were as follows: 15 minutes initial denaturation at 95°C, then 40 cycles of 15 sec 95°C, 1 min at 56°C, 30 sec at 72°C. Primers sequences used were reported in Payyavula et al. [16].

## Results

### Water Utilization and Biomass Accumulation

**Figure 1:**
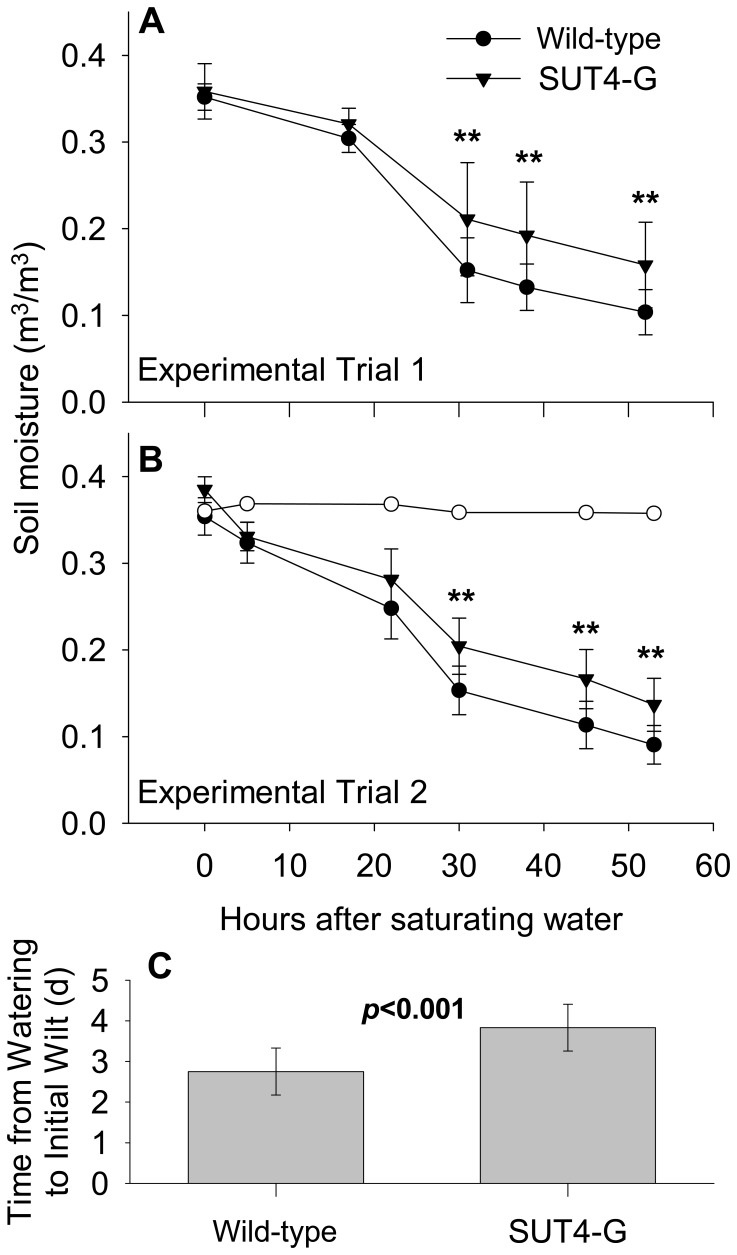
*Populus* water uptake during acute drought stress. Differences in plant water uptake were estimated by comparing the rates of soil water loss. Data is shown for two experimental trials (A) and (B). Data points represent means ± SD. Concurrent soil moisture changes in plant-free pots (o) are included in (B). Each experimental trial included eight wild-type and six SUT4-RNAi plants. (C) Onset of wilt symptoms averaged from the two experimental trials. **p≤0.01 as determined by Student’s *t*-test.

In the acute drought experiment, water was withheld from potted plants until the onset of leaf turgor loss. Plant water uptake assessed by the rate of SRWC decrease preceding turgor loss was slower in RNAi plants (Figure 1A–B). In addition, the onset of turgor loss occurred one day later in RNAi than wild-type plants (Figure 1C). The SRWC change in plant-free pots was negligible (Figure 1B). Significant changes in growth metrics were not expected to occur as a result of the short, acute treatment. The SUT4-RNAi and wild-type plants used for the experiment exhibited similar average height growth increments of approximately 2.5–3 cm/day, depending on the cohort (Table 1). Total leaf area, leaf:root ratio, and the ratio of stem diameter to height were largest in SUT4-RNAi plants (Table 1). Stem mass was comparable between SUT4-RNAi and wild-type plants, but water comprised a higher percentage of fresh stem weight in SUT4-RNAi plants (Table 1).

**Table 1:** Biomass data for two independent cohorts of wild-type and transgenic plants used in acute drought experiments.

	Trial 1						Trial 2						Mean					
	WT			SUT4-G			WT			SUT4-G			WT			SUT4-G		
Height (cm)	**104.02±8.30**			**93.36±8.14**			137.51±9.44			132.68±6.22			**121.88±19.13**			**109.45±21.07**		
Diameter (mm)																		
LPI 5	6.11±0.40			5.93±0.47			5.90±0.25			6.12±0.31			6.00±0.34			6.01±0.42		
LPI 20	9.97±1.03			9.31±0.94			9.42±0.66			9.63±0.44			9.68±0.88			9.44±0.78		
Base	10.04±0.89			9.63±0.90			11.77±0.82			12.29±0.87			10.96±1.21			10.72±1.59		
Height:Diameter Ratio	**10.39±0.68**			**9.71±0.56**			**11.69±0.45**			**10.73±0.84**			**11.08±0.87**			**10.07±0.82**		
Height Growth Rate (cm/day)	2.89±0.21			3.02±0.22			2.49±0.22			2.42±0.26			2.68±0.30			2.77±0.38		
Leaf Area (cm^2^)																		
LPI 6	**199.70±38.74**			**230.72** ***±*** **37.70**			*175.76±28.34*			*206.81±55.23*			**187.31±35.31**			**222.36±44.70**		
LPI 15	**316.62±40.05**			**366.86±57.48**			373.06±68.18			400.41±60.59			*346.72±62.80*			*378.60±59.29*		
Tissue Dry Mass (g)																		
Total Aboveground	125.51±35.23			114.42±33.95			123.51±13.00			132.58±25.02			124.36±23.86			119.87±31.38		
Leaf	48.25±10.69			45.86±11.39			*45.67±5.64*			*52.29±1.75*			46.78±7.92			47.79±9.84		
Stem	77.26±25.62			68.56±23.21			77.84±10.08			80.29±23.37			77.59±17.53			72.08±22.64		
Wood (% of Stem dry mass)	**74.54±1.15**			**73.14±0.46**			74.01±0.83			73.26±2.25			**74.24±0.98**			**73.18±1.12**		
Bark (% of Stem dry mass)	**25.46±1.15**			**26.86±0.46**			25.99±0.83			26.74±2.25			**25.76±0.98**			**26.82±1.12**		
Root	14.17±5.91			11.82±3.62			15.85±3.18			12.89±1.80			*15.13±4.43*			*12.14±3.12*		
Tissue Mass Ratio																		
Leaf:Stem	0.65±0.11			0.69±0.11			0.59±0.09			0.68±0.16			0.62±0.10			0.69±0.12		
Leaf:Root	3.64±0.79			3.99±0.92			**2.99±0.74**			**4.10±0.46**			**3.27±0.80**			**4.02±0.78**		
Stem:Root	5.67±0.94			5.92±1.81			5.04±0.89			6.16±1.03			5.31±0.93			5.99±1.56		
Shoot:Root	9.30±1.54			9.91±2.66			**8.03±1.53**			**10.25±0.79**			*8.58±1.61*			*10.01±2.21*		
Water Concentration (% FW)																		
Wood	**72.2±1.48**			**74.22±0.82**			**72.85** ***±*** **1.20**			**75.53** ***±*** **2.90**			**72.57±1.31**			**74.61±1.65**		
Bark	80.23±0.81			80.34±1.28			79.19±0.99			79.20±4.43			79.63±1.03			80.00±2.40		

Values are means ± SD. Bold values represent *p*≤0.05 and italicized values represent 0.05<*p*<0.1, as determined by Student's *t*-test.

**Figure 2:**
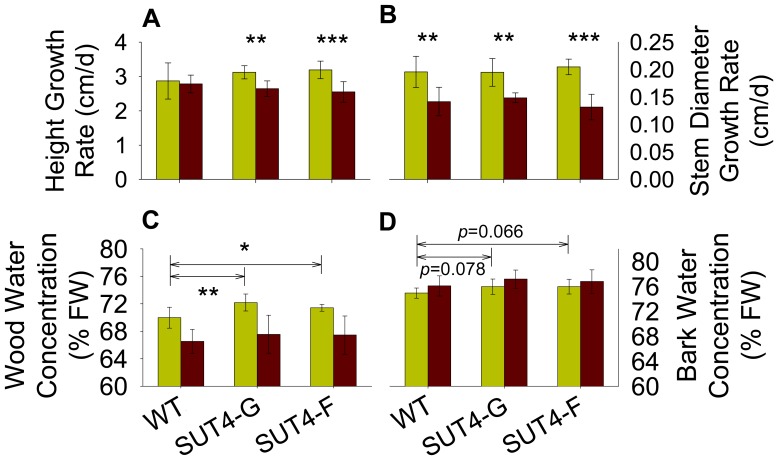
Effects of water availability on *Populus* stem growth and water concentrations. (A) height growth rate, (B) stem diameter growth rate, (C) wood water concentrations (g/g×100), and (D) bark water concentration (g/g×100). Light and dark bars represent high and low soil moisture, respectively. For (A) and (B), asterisk denotes statistical significance between high and low soil moisture within a line. For (C) and (D), asterisks and *p*-values represent pairwise comparisons between high soil moisture groups delineated by the arrows (i.e., wild type vs. one of the transgenic lines). Bars represent means ± SD of 6–8 replicate plants. *p≤0.05, **0.001<*p*≤0.01, ***p≤0.001 as determined by Student’s *t*-test.

For the chronic, less severe drought treatment, plants were maintained for two months under well-watered versus water-limiting conditions. In general, RNAi plants were more sensitive than wild-type plants to prolonged differences in SRWC. Leaf areas were higher in SUT4-RNAi than wild-type plants under well-watered conditions, but suffered greater decreases under water-limiting conditions (Table 2). Leaf water concentrations (% dry mass basis) were lower in SUT4-RNAi than wild-type plants with reduced SRWC, but specific leaf dry mass was unaltered by SUT4 perturbation or water regime (Table 2). Furthermore, height growth was most reduced under low SRWC in SUT4-RNAi plants (Figure 2A), while diameter growth rate was reduced under low SRWC in all plants (Figure 2B). Wood and bark water concentrations were higher in well-watered SUT-RNAi plants than in wild-type, but no difference between genotypes was observed under low SRWC (Figure 2C–D).

**Table 2:** Area, water content, and specific leaf area of source leaves (LPI 10) from wild-type and transgenic *Populus* under contrasting long-term soil moisture regimes.

		WT	SUT4-G	SUT4-F	*p*WT vs. G	*p*WT vs. F
Area	High soil moisture	247.8±40.9	287.3±29.9	296.1±39.5	*0.055*	**0.037**
(cm^2^)	Low soil moisture	254.0±25.7	231.9±35.2	247.8±56.7	0.236	0.796
	*p* (high vs. low)	0.738	**0.015**	*0.089*		
Water content	High soil moisture	68.62±1.38	69.34±2.03	69.32±2.08	0.443	0.444
(% FW)	Low soil moisture	69.92±1.68	67.90±1.21	68.25±1.26	**0.045**	*0.057*
	*p* (high vs. low)	0.124	0.125	0.478		
Specific leaf area	High soil moisture	245.3±19.2	260.9±38.1	237.0±12.4	0.326	0.348
(cm^2^/g DW)	Low soil moisture	255.6±33.3	248.0±14.8	259.8±18.1	0.646	0.776
	*p* (high vs. low)	0.467	0.495	**0.018**		

Values are means ± SD of n = 6–8 replicates per treatment group. Bold values are *p<*0.05, italics are 0.05<*p*<0.1 as determined by Student’s *t-*test.

### Leaf Gas Exchange and Chlorophyll Concentration

**Figure 3:**
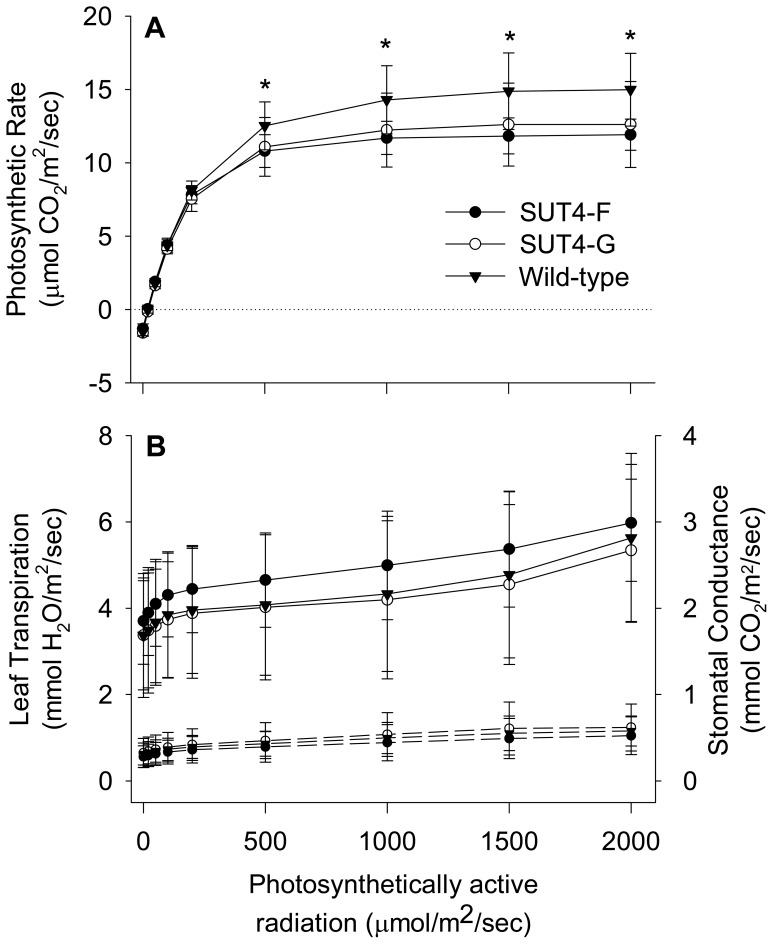
Leaf gas exchange properties of *Populus* wild-type and RNAi plants. Light response curves for (A) Photosynthetic CO_2_ fixation, and (B) Leaf transpiration (solid lines) and stomatal conductance (dashed lines). A fully expanded source leaf (LPI-10) was used for the measurements. Data points are means ± SD of 19 WT, 10 line G, and 12 line F plants. * *p*≤0.05 for each transgenic line compared to wild type as determined by Student’s *t*-test.

Basal respiration, quantum yield and light compensation point were similar between well-watered SUT4-RNAi and wild-type plants used in the acute drought experiment, but photosynthesis (A_max_) was reduced in the SUT4-RNAi plants (Table 3). A_max_ was also lower in well-watered SUT4-RNAi plants than wild-type plants in the chronic drought experiment (Table 4; Figure 3A). A_max_ increased under water-limiting conditions in the SUT4-RNAi plants, but was stable across moisture regimes in wild-type plants (Figure 3A; Table 4). C_i_ varied as expected in accordance with changes in A_max_ due to carboxylation limitation, and was highest when A_max_ was lowest (Table 4). In contrast, Gs and E were not different between RNAi and wild-type leaves under normal watering conditions, and decreased similarly in all plants during water-limited growth (Figure 3B; Table 4). Concentrations of chlorophyll a, chlorophyll b, and their ratio were also not affected by SUT 4 perturbation, though they all were responsive to soil moisture availability (Table 4, Figure S3).

**Table 3:** Gas exchange in source leaves (LPI 15) of wild-type and transgenic *Populus* from the chronic drought experiment.

		WT	SUT4-G	SUT4-F	*p* 5WT vs. G	*p*WT vs. F
		mean±SD	mean±SD	mean±SD		
Amax^1^	High soil moisture	20.08±1.71	16.37±1.97	18.67±1.11	**0.004**	*0.100*
(µmol/m^2^/s)	Low soil moisture	19.47±1.30	19.78±1.01	21.36±1.58	0.674	**0.031**
*p* 5 (high vs. low)		0.485	**0.006**	**0.003**		
Conductance	High soil moisture	0.91±0.17	0.92±0.08	0.76±0.15	0.972	0.116
(mmol/m^2^/s)	Low soil moisture	0.73±0.09	0.61±0.09	0.80±0.12	**0.033**	0.233
	*p* (high vs. low)	**0.032**	**<0.001**	*0.059*		
Transpiration	High soil moisture	8.35±0.97	8.72±0.49	7.47±1.02	0.396	0.141
(mmol/m^2^/s)	Low soil moisture	7.74±0.92	6.95±0.87	7.99±0.79	0.165	0.599
	*p* (high vs. low)	0.268	**0.001**	0.310		
Ci (ppm)	High soil moisture	309.2±7.2	331.5±17.9	324.1±7.9	**0.016**	**0.005**
	Low soil moisture	315.4±12.1	308.9±21.8	313.0±13.1	0.503	0.728
	*p* (high vs. low)	0.299	*0.073*	*0.078*		
Photosynthetic WUE 2	High soil moisture	2.42±0.25	1.88±0.24	2.54±0.36	**0.002**	0.521
(mmol/mol)	Low soil moisture	2.56±0.46	2.88±0.40	2.70±0.35	0.237	0.540
	*p* (high vs. low)	0.523	**<0.001**	0.411		
Chlorophyll a^3^	High soil moisture	0.11±0.02	0.10±0.02	0.12±0.02	0.364	0.699
(nmol/mg DW)	Low soil moisture	0.14±0.01	0.14±0.01	0.15±0.01	0.539	0.603
	*p* (high vs. low)	**0.002**	**0.002**	**0.010**		
Chlorophyll a^4^	High soil moisture	0.46±0.10	0.40±0.07	0.48±0.10	0.227	0.852
(nmol/cm^2^)	Low soil moisture	0.57±0.08	0.57±0.04	0.56±0.05	0.753	0.683
	*p* (high vs. low)	**0.030**	**0.001**	*0.064*		
Chlorophyll b	High soil moisture	0.09±0.02	0.09±0.02	0.10±0.03	0.421	0.658
(nmol/mg DW)	Low soil moisture	0.13±0.01	0.13±0.01	0.14±0.02	0.495	0.626
	*p* (high vs. low)	**0.003**	**0.002**	**0.015**		
Chlorophyll b	High soil moisture	0.39±0.11	0.33±0.07	0.41±0.10	0.289	0.718
(nmol/cm^2^)	Low soil moisture	0.52±0.09	0.50±0.05	0.51±0.05	0.715	0.830
	*p* (high vs. low)	**0.021**	**0.001**	**0.037**		
Chl a/Chl b	High soil moisture	1.20±0.06	1.21±0.05	1.18±0.08	0.744	0.598
	Low soil moisture	1.11±0.06	1.13±0.04	1.10±0.06	0.487	0.714
	*p* (high vs. low)	**0.009**	**0.008**	**0.043**		

1 Determined at saturating light intensity of 1500 µmol/m^2^/sec.

2 WUE = A_max_/Transpiration.

3 Chlorophyll concentrations determined using equations presented in Porra et al. 1989.

4 Specific leaf area calculations were used to present the Chl data per unit leaf area.

5 Determined by Student’s *t-*test.

**Table 4:** Comparison of gas exchange parameters among well-watered wild-type and transgenic *Populus* used in the acute drought experiment.

	WT	SUT4-G	*p* 1	SUT4-F	*p*
A_max_ (mmol/m^2^/s)	**14.62±2.43**	**12.48±2.75**	**0.025**	**11.81±1.11**	**0.001**
Conductance (mmol/m^2^/s)	0.54±0.20	0.61±0.31	0.360	0.52±0.24	0.673
Transpiration (mmol/m^2^/s)	4.77±1.34	5.37±1.92	0.241	4.55±1.85	0.653
Respiration (mmol/m^2^/s)	−1.47±0.31	−1.59±0.24	0.401	−1.31±0.32	0.087
Quantum yield (mol CO_2_/mol PAR^2^)	0.06±0.00	0.06±0.00	0.316	0.06±0.00	0.378
Light compensation point (mmol/m^2^/s PAR)	21.98±4.38	24.60±2.72	0.106	20.38±4.00	0.164
Photosynthetic WUE^3^ (mmol CO_2_/mol H_2_O)	3.34±1.08	2.75±1.28	0.173	2.90±1.17	0.321
Ci = Intracellular [CO_2_] (ppm)	316.19±34.01	320.69±23.60	0.644	318.73±32.16	0.814

1 *P*-values as determined by Student’s *t-*test represent comparisons between the specific SUT4-RNAi line and the wild type, based on means ± SD of 19 WT, 10 SUT4-G and 12 SUT4-F plants.

2 PAR = Photosynthetically active radiation.

3 WUE = Water use efficiency = A_max_/Respiration.

### Foliar Suc and RFO Responses to Contrasting SRWC

**Figure 4:**
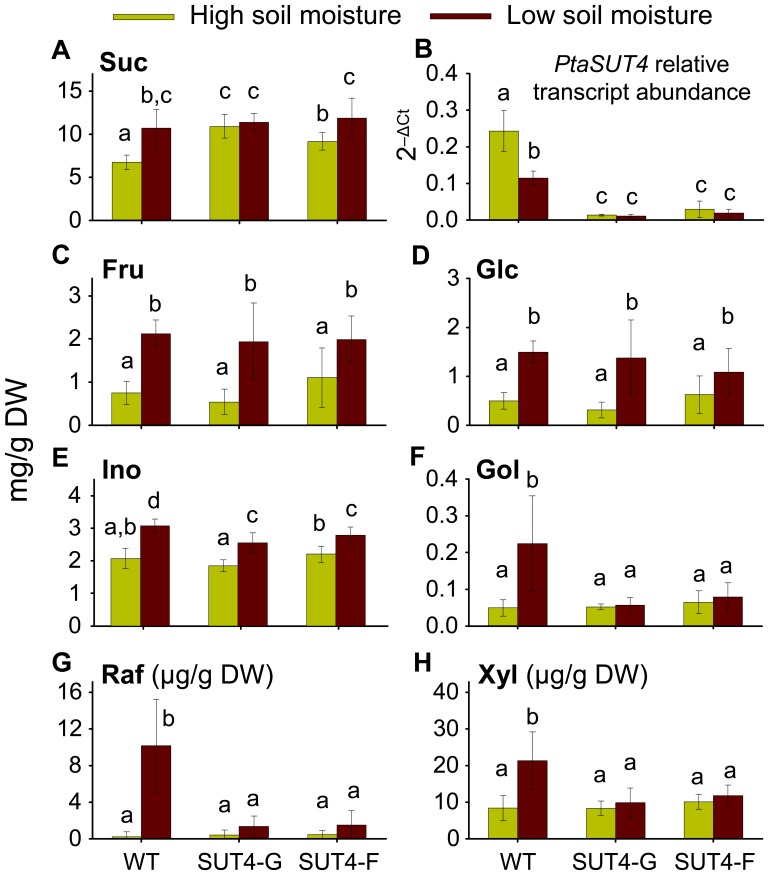
Effects of water availability on sucrose and RFO metabolism in source leaves. Metabolite concentrations (mg/g DW in A-F or µg/g DW in G-H) were determined using standard curves of authentic standards. Light and dark bars represent plants maintained under high or low soil moisture, respectively. Bars are means ± SD of 6–8 replicates each. Bars with different letters are statistically different at α = 0.05 based on Student’s *t* post-hoc test of LSMeans models using JMP 9.0 (SAS Institute, Cary, NC).

Suc concentrations were higher in mature leaves, xylem and phloem/bark tissues of SUT4-RNAi plants than wild type under well-watered conditions (Figure 4A; Table S1). Under chronic water-limiting conditions, leaf expression of *PtaSUT4* was substantially down-regulated in wild-type plants (Figure 4B), and leaf Suc concentrations increased by 59%, nearly matching levels observed in leaves of the RNAi plants (Figure 4A). Fru and Glc also responded strongly to water-limiting conditions independent of SUT4 expression (Figure 4C–D). Ino (an RFO precursor derived from Glc [38]) concentrations were more strongly induced in wild type than SUT4-RNAi plants experiencing water-limitation (Figure 4E). Under the same conditions, xylitol and the RFOs Gol and Raf, were sharply up-regulated in wild type, but not SUT4-RNAi plants (Figure 4F-H). Furthermore, Ino/Glc ratios were not affected by SUT4 perturbation, but Gol/Ino and Raf/Gol were both lower in SUT4-RNAi plants relative to wild types under water-limiting conditions (Table 5).

**Table 5:** Metabolite ratios of source leaves (LPI 15) of wild-type and transgenic *Populus.*

		WT	SUT4-G	SUT4-F	*p* 1 WT vs. G	*p* WT vs. F
		**mean±SD**	**mean±SD**	**mean±SD**		
Suc/Fru	High soil moisture	9.83±2.82	28.02±21.19	11.35±7.84	**0.031**	0.615
	Low soil moisture	5.14±1.42	8.33±6.99	6.32±1.81	0.261	0.200
	*p* 1 (high vs. low)	**0.002**	**0.037**	0.124		
Suc/Glc	High soil moisture	14.64±4.37	44.79±26.25	19.43±11.97	**0.007**	0.309
	Low soil moisture	7.29±1.88	14.19±13.53	12.32±4.86	0.206	**0.025**
	*p* (high vs. low)	**0.001**	**0.018**	*0.171*		
Fru/Glc	High soil moisture	1.51±0.21	1.71±0.29	1.75±0.15	0.132	**0.024**
	Low soil moisture	1.43±0.14	1.59±0.46	1.91±0.31	0.389	**0.003**
	*p* (high vs. low)	0.424	0.567	0.251		
Ino/Glc	High soil moisture	4.48±1.36	7.24±3.59	4.42±1.87	*0.064*	0.941
	Low soil moisture	2.03±0.38	2.82±2.11	2.93±1.20	0.387	0.108
	*p* (high vs. low)	**0.001**	**0.016**	*0.100*		
Gol/Ino	High soil moisture	0.021±0.004	0.028±0.004	0.029±0.011	***0.*** **006**	0.109
	Low soil moisture	0.058±0.021	0.022±0.008	0.028±0.013	***0.*** **002**	**0.021**
	*p* (high vs. low)	**0.001**	*0.075*	0.954		
Raf/Gol	High soil moisture	0.0012±0.0030	0.0068±0.0100	0.0064±0.0070	0.179	0.094
	Low soil moisture	0.0555±0.0210	0.0240±0.0240	0.0103±0.0120	**0.042**	**0.002**
	*p* (high vs. low)	**<0.001**	0.106	0.484		

1 Pairwise comparisons were conducted using Student’s *t-*test.

## Discussion

The experiments reported here extend previous findings that RNAi down-regulation of a tonoplastic SUT, *PtaSUT4*, led to increased Suc levels, increased vacuolar sequestration of Suc, and altered expression of genes related to carbon partitioning in *Populus* plants [16]. Here, we investigated the role of PtaSUT4 in shoot water relations and gas exchange. The general finding was that *PtaSUT4-*suppression altered water uptake as well as the physiological responsiveness of the transgenics to water availability and drought stress. A_max_, C_i_, area-corrected leaf water content, height, and stem water content all changed in response to chronic water deficit in SUT4-RNAi but not wild-type plants. Further, induction of Gol and Raf during chronic water deficit was only observed in wild-type plants.

We observed that water uptake by RNAi plants was intrinsically slower than by wild-type plants upon water withholding (Figure 1). A related finding from both the acute and chronic drought experiments was that in the absence of stress (well-watered controls), the water content of stem xylem (wood) was higher in RNAi than wild-type plants (Table 1, Figure 2C). Although specific leaf water content was not higher in RNAi plants, leaf area was greater in both experiments (Tables 1 and 2). Therefore, it appears that increased sequestration of Suc within leaf vacuoles promoted increased leaf expansion while altering normal uptake and movement of water through the conducting xylem. This is consistent with the reported inverse correlation between the concentration of polar metabolites in leaf tissues and the hydraulic conductance of the vascular system [39]. Furthermore, our findings suggest that the relationship between hydraulic conductivity and leaf osmolarity may also depend on subcellular distribution of metabolites like Suc.

In the acute drought experiment where plants did not have sufficient time to acclimate to the stress, transgenic down-regulation of *PtaSUT4* appears to have potentiated at least a transient tolerance of RNAi plants to soil drying (Figure 1C). In fact, source leaf expression of *PtaSUT4* did not change in the more susceptible wild-type plants during the 3 day time frame of the acute drought experiment (data not shown). During the chronic, non-lethal drought experiment, *PtaSUT4* expression decreased, and Suc levels increased in the wild-type plants (Figure 4A). Although the decrease in *PtaSUT4* transcript levels did not quite mimic the transcript level reduction in the RNAi plants, they were reduced by 50%, and Suc levels increased nearly 60% to match the levels of the RNAi plants. From the two experiments, it appears that vacuolar sequestration of Suc forms part of the natural adaptive response to changes in plant water status. Vacuolar sequestration of Suc also appears to reduce transpirational losses and soil water uptake, although those effects were only possible to attribute to PtaSUT4 using plants with artificial down-regulation. Based on these findings as well as the effects on biomass allocation that were also described, regulation of *PtaSUT4* potentially integrates the utilization of water and carbon with soil water availability in *Populus*.

From our earlier work with the PtaSUT4-RNAi plants [16], the constitutive sequestration of Suc in source leaf vacuoles ultimately reduced the supply of Suc to newly expanding leaves. Based on that work and the present findings, the potential exists for a trade-off between the provisioning of terminal sinks, and the vacuolar sequestration of Suc during drought-like conditions. This places PtaSUT4 at a potential crossroads between the isohydric and anisohydric general responses to drought as put forth by Tardieu [40]. During the isohydric response to water deficit, plants maintain leaf water potential by lowering stomatal conductance. The potential drawback to this strategy is carbon starvation [41]. A wide range of *Populus* genotypes exhibit a comparatively isohydric response of decreased stomatal conductance and photosynthesis during water deficits [42]–[44]. Comparatively drought tolerant *Populus* genotypes exhibit a more anisohydric response in which leaf water potential decreases and stomates remain partially open during drought [44]. In addition, isohydric responses have been described in drought tolerant *Populus* that apparently maintain carbon flux into the root system [45], [46]. This suggests additional feedback loops between sink carbon demand, source photosynthetic capacity, and sustained solute flow during the acquisition of drought tolerance [45], [46]. Tonoplast trafficking mediated by SUT4 offers a strategy in such feedback loops by which vacuolar sequestration of Suc can minimize feedback inhibition of photosynthesis by Suc, while enabling an (anisohydric) decrease in cell water potential and sustaining partial stomatal conductance. This is consistent with the sustained A_max_ and elevated leaf sucrose contents that were observed in wild-type plants during the chronic drought treatment. As illustrated in the short-term acute drought experiment, the timing of *PtsSUT4* expression changes may be critical to the orchestration of these adaptive events during drought onset. Exacerbating effects of high temperature on the rate of drought onset and, potentially on *PtaSUT4* regulation, could therefore mitigate or enhance *Populus* drought acclimation in future environments.

The RNAi suppression of *PtaSUT4* had a negative effect on the induction of RFOs, suggesting that the natural regulation of *PtaSUT4* is important for balancing Suc compartmentalization in a physiologically beneficial way. RFOs are involved in abiotic and biotic stress responses [38], [47]–[54], and are synthesized in the cytosol from Suc and other precursors [38], [55], [56]. Therefore, RFO induction during drought implies a continuing cytosolic demand for Suc which apparently is met in wild-type plants despite partial down-regulation of *PtaSUT4*. In addition, decreased ratios of Gol/Ino but not Ino/Glc in SUT4-RNAi plants in water-limited conditions (Table 5) suggest that SUT4 activity affects Gol synthase activity. Interestingly, stress-responsive Gol synthase gene expression and Suc accumulation occur together in DREB1-overexpressing *Arabidopsis* or tobacco [50], [57], [58]. In light of the findings from the acute and chronic drought experiments, constitutive hyper-sequestration of Suc in the vacuole of RNAi plants may potentiate drought tolerance in un-acclimated plants while compromising RFO synthesis in the cytosol of plants that are adapting to chronic water limitation. Wingenter et al. [59] found that overexpressing the tonoplastic monosaccharide transporter 1 (an antiporter which transports hexoses as well as Suc into the vacuole [60]) reduced the sensitivity of sugar signaling mechanisms when exposed to exogenous Suc. This suggests an alternative, or a partner to cytosolic starvation as an explanation for the attenuated/blocked synthesis of RFOs in the drought-stressed RNAi plants. In effect, Suc may have been hidden from cytosolic sugar sensing mechanisms by the vacuolar sequestration of Suc in RNAi plants.

A remaining question concerns the A_max_ reduction observed in source leaves of water-replete RNAi plants (Tables 3 and 4). Elevated sucrose can lead to photosynthetic inhibition in apoplastic loading herbaceous species, many of which normally maintain low sucrose levels in source leaf mesophyll [28]. Although elevated Suc might impose a negative feedback on the Calvin-Benson cycle [61], [62] or sugar signaling networks [63], [64], negative effects on photosynthesis were not observed in chronically water-stressed wild-type or RNAi plants even though Suc levels actually increased (Table 4; Figure 4). Eom et al. [22] reported no effect on photosynthesis following RNAi suppression of the rice Group IV tonoplast *OsSUT2* despite comparable increases in leaf Suc concentrations. In fact, *Populus* and other Salicaceae that grow in temperate climates maintain relatively high concentrations of source leaf Suc in comparison to apoplastic loading herbaceous species [28], [65]. Their photosynthesis may therefore be inherently less sensitive to shifts in Suc level, at least when they are grown in natural light under carbon non-limiting conditions. One alternative explanation to Suc inhibition is that light harvesting itself was compromised in the leaves of well-watered RNAi plants. However, neither Chl concentration nor specific leaf area were reduced in well-watered RNAi plants (Table 2). These argue against a dilution of the photosynthetic machinery per unit leaf area in the water-replete SUT4-RNAi plants. However, the vacuole plays a role in cellular distribution of chloroplasts to optimize light capture [66]. The importance of chloroplast orientation to light capture has been well documented [67], [68] and, in the present context, the possibility remains that increases in vacuole dimensions somehow perturbed chloroplast packing, orientation, or movements in well-watered RNAi plants, compromising light harvesting efficiency.

As a final point, SUT4 regulation of tissue water balance and responses to water stress in *Populus* may have practical application. Poplars are important in biomass plantations [69], and transgenesis may be a useful strategy for tree improvement in some cases [70]. Our results illustrate the potential for altered PtaSUT4 activity to have an impact on drought-responsive growth and performance characteristics of this economically important genus.

## Supporting Information

Figure S1 **Estimation of wilting point.** Initial tests of (A) soil dry-down timing and (B) wilting point estimation in the experimental *Populus* clone.
(PDF)

Figure S2 **Representative photograph used for calculating leaf area.** Leaf areas were determined by photographing leaves against a white background containing three standard areas. All areas were then determined using SigmaScan 3.5 relative to the standard curve for each photograph (See Materials and Methods). The average and lowest R^2^ for a standard curves were 0.998 and 0.987, respectively.
(PDF)

Figure S3 **Spectral analysis of ethanol extracts of** ***Populus*** **source leaves.** Spectra of LPI 15 mature leaf extracts from 200–800 nm from (A) Wild type, (B) SUT4-G, and (C) SUT4-F leaves. The inset visible spectrum aligns with the wavelengths. Arrows in the top graph correspond to the maximum absorbances of Chlorophyll a, 430 nm and 660 nm. (D) Photograph of leaves from high and low soil moisture regimes. Photograph shows leaves grown under water-limiting conditions that were typically smaller with a darker shade of green relative to leaves from plants grown in well-watered conditions.
(PDF)

Table S1 Sucrose concentrations (mg/g DW) in the bark and xylem of wild-type and transgenic *Populus* under standard watering regimes.
(PDF)
