# Supplementary material for: The Tonoplast-Localized Sucrose Transporter in Populus (PtaSUT4) Regulates Whole-Plant Water Relations, Responses to Water Stress, and Photosynthesis
Source: PLoS One. 2012 Aug 31;7(8):e44467. doi: 10.1371/journal.pone.0044467 (PMC3432113; doi:10.1371/journal.pone.0044467)
Supplement: Figure S1 — Estimation of wilting point. Initial tests of (A) soil dry-down timing and (B) wilting point estimation in the experimental Populus clone. (PDF) [file pone.0044467.s001.pdf]

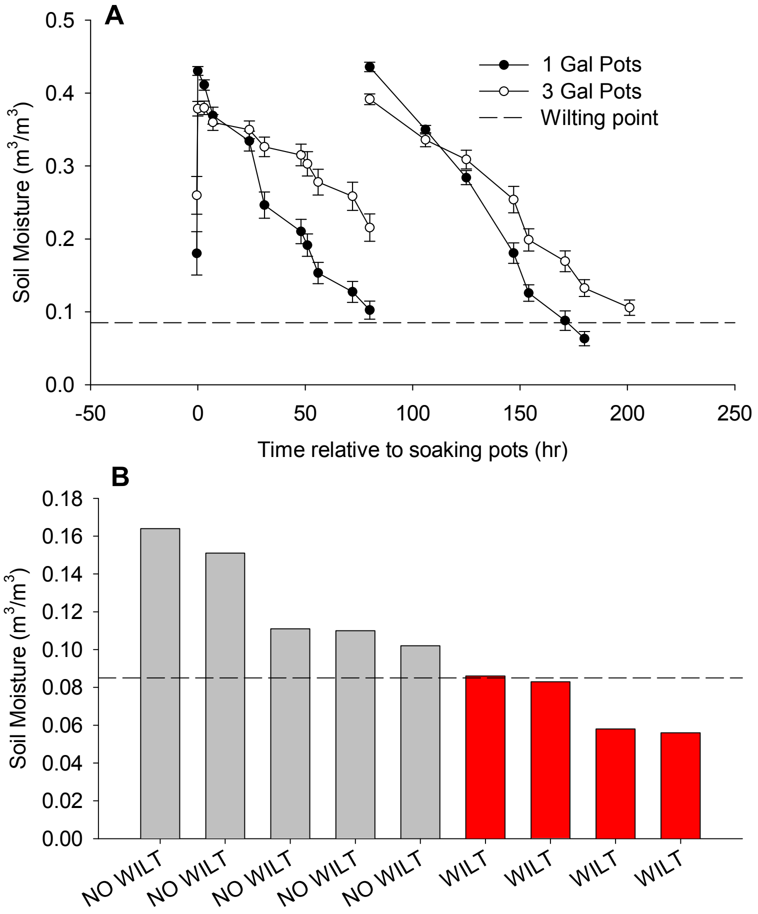

Figure S1. Estimation of wilting point.

Initial tests of (A) soil dry-down timing and (B) wilting point estimation in the experimental *Populus* clone.
