# Supplementary material for: The Tonoplast-Localized Sucrose Transporter in Populus (PtaSUT4) Regulates Whole-Plant Water Relations, Responses to Water Stress, and Photosynthesis
Source: PLoS One. 2012 Aug 31;7(8):e44467. doi: 10.1371/journal.pone.0044467 (PMC3432113; doi:10.1371/journal.pone.0044467)
Supplement: Figure S2 — Representative photograph used for calculating leaf area. Leaf areas were determined by photographing leaves against a white background containing three standard areas. All areas were then determined using SigmaScan 3.5 relative to the standard curve for each photograph (See Materials and Methods). The average and lowest R2 for a standard curves were 0.998 and 0.987, respectively. (PDF) [file pone.0044467.s002.pdf]

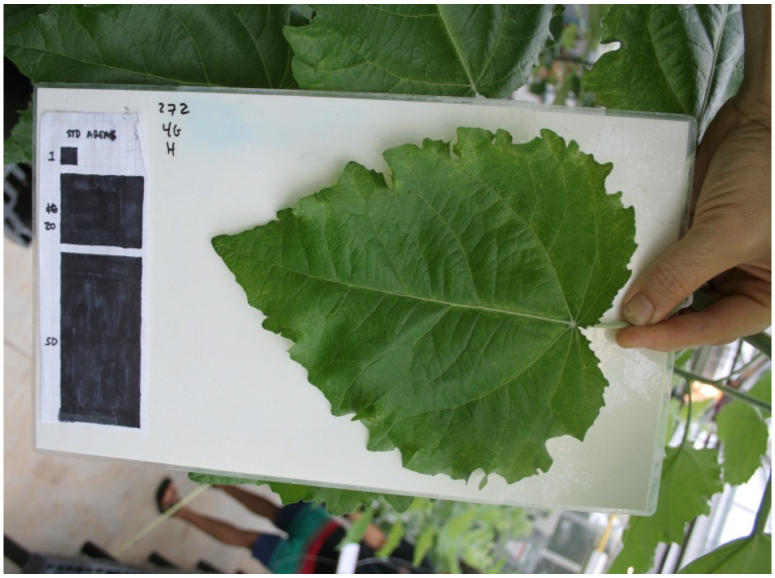

Figure S2. Representative photograph used for calculating leaf area. Leaf areas were determined by photographing leaves against a white background containing three standard areas. All areas were then determined using SigmaScan 3.5 relative to the standard curve for each photograph (See Methods). The average and lowest R^2 for a standard curves were 0.998 and 0.987, respectively.
