# Supplementary material for: The Tonoplast-Localized Sucrose Transporter in Populus (PtaSUT4) Regulates Whole-Plant Water Relations, Responses to Water Stress, and Photosynthesis
Source: PLoS One. 2012 Aug 31;7(8):e44467. doi: 10.1371/journal.pone.0044467 (PMC3432113; doi:10.1371/journal.pone.0044467)
Supplement: Figure S3 — Spectral analysis of ethanol extracts of Populus source leaves. Spectra of LPI 15 mature leaf extracts from 200–800 nm from (A) Wild type, (B) SUT4-G, and (C) SUT4-F leaves. The inset visible spectrum aligns with the wavelengths. Arrows in the top graph correspond to the maximum absorbances of Chlorophyll a, 430 nm and 660 nm. (D) Photograph of leaves from high and low soil moisture regimes. Photograph shows leaves grown under water-limiting conditions that were typically smaller with a darker shade of green relative to leaves from plants grown in well-watered conditions. (PDF) [file pone.0044467.s003.pdf]

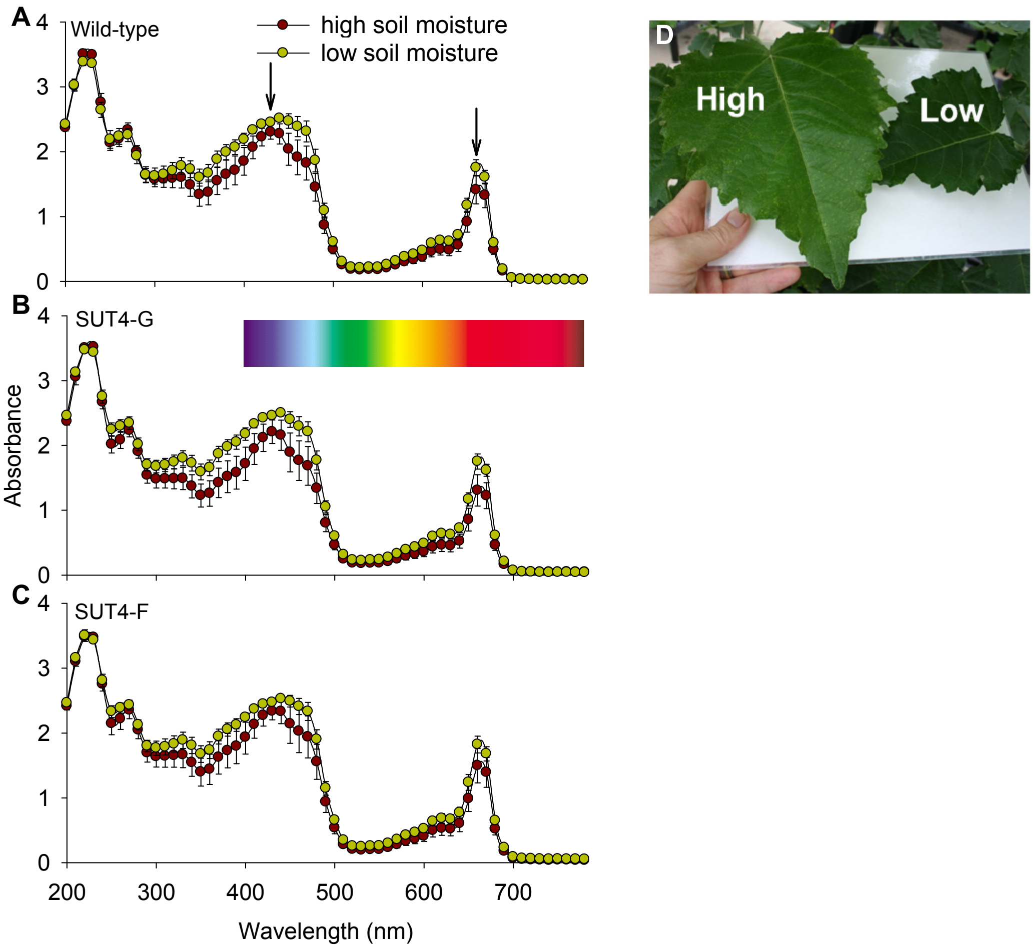

Figure S3. Spectral analysis of ethanol extracts of *Populus* source leaves.
Spectra of LPI 15 mature leaf extracts from 200-800 nm from (A) Wild type, (B) SUT4-G, and (C) SUT4-F leaves. The inset visible spectrum aligns with the wavelengths. Arrows in the top graph correspond to the maximum absorbances of Chlorophyll a, 430nm and 660nm. (D) Photograph of leaves from high and low soil moisture regimes. Photograph shows leaves grown under water-limiting conditions that were typically smaller with a darker shade of green relative to leaves from plants grown in well-watered conditions.
