# Supplementary material for: The Tonoplast-Localized Sucrose Transporter in Populus (PtaSUT4) Regulates Whole-Plant Water Relations, Responses to Water Stress, and Photosynthesis
Source: PLoS One. 2012 Aug 31;7(8):e44467. doi: 10.1371/journal.pone.0044467 (PMC3432113; doi:10.1371/journal.pone.0044467)
Supplement: Table S1 — Sucrose concentrations (mg/g DW) in the bark and xylem of wild-type and transgenic Populus under standard watering regimes. (PDF) [file pone.0044467.s004.pdf]

Table S1. Sucrose concentrations (mg/g DW) in the bark and xylem of wild-type and transgenic *Populus* under standard watering regimes.

	**Bark**	**Xylem**
WT	8.806 ± 1.382	8.452 ± 2.386
SUT4-G	11.166 ± 1.535	12.306 ± 3.441
p value	**<0.001**	**0.005**

Values are means ± SD. Pairwise comparisons were conducted using Student's *t*-test
